# Approval lag and loss for biologic-device combination products in Japan and the United States

**DOI:** 10.3389/fmedt.2026.1924169

**Published:** 2026-09-18

**Authors:** Makiko Mochizuki, Hideki Maeda

**Affiliations:** 1 Department of Regulatory Science, Graduate School of Pharmaceutical Sciences, Meiji Pharmaceutical University, Tokyo, Japan; 2 MSD K.K., Tokyo, Japan

**Keywords:** autoinjectors, combination products, drug delivery devices, drug-device combination products, Japan, medical devices, prefilled syringes

## Abstract

**Introduction:**

The market for drug delivery devices (DDDs), including combination products that integrate pharmaceuticals and medical devices, has expanded rapidly with the increasing use of biopharmaceuticals and growing adoption of self-administration. However, the extent and causes of approval lag and approval loss for device presentations, defined as presentations approved in one country but unavailable in the other, have not been systematically evaluated between Japan and the United States.

**Methods:**

We analyzed approvals of biopharmaceuticals in various device presentations, including prefilled syringes (PFS), autoinjectors, multidose pen injectors, on-body injectors (OBIs), and cartridge formulations, using the Pharmaceuticals and Medical Devices Agency (PMDA) and U.S. Food and Drug Administration (FDA) databases from 2000 to 2025.

**Results:**

Among 176 biopharmaceutical products approved in the United States, 16% were not approved in Japan. However, most cases were attributable to approval loss by drug, reflecting products whose active substances were not approved for the Japanese market, rather than approval loss by device presentation. Although median approval lag tended to vary across device presentations, with multidose pen injectors and cartridge presentations showing median delays exceeding approximately two years and autoinjectors showing the shortest lag at approximately 10 months, these differences were not statistically significant. Statistical analyses using Pearson's chi-square, Fisher's exact, and Kruskal–Wallis tests showed no significant differences in device presentation approval between Japan and the United States after excluding products lacking active substance approval in either country (*p* = 0.961 and 0.963), or in approval lag among device presentations in Japan-first and U.S.-first products (*p* = 0.088 and 0.299).

**Discussion:**

In the United States, multiple device presentations were often approved simultaneously, whereas Japan more frequently adopted a stepwise approval approach. Differences in the regulatory scope of combination products and co-packaging requirements also contributed to country- specific packaging, potentially prolonging pre-approval preparation and increasing supply chain complexity. Overall, regulatory approval discrepancies between Japan and the United States were driven primarily by drug loss rather than device presentation loss. Accelerating drug approval, promoting simultaneous development of multiple device presentations, and enhancing regulatory harmonization for combination products may reduce approval lag by device presentation and improve patient access.

## 1 Introduction

Over the past two decades, the time gap between drug approvals by the U.S. FDA or the European Medicines Agency (EMA) and subsequent approvals in Japan, commonly referred to as “drug lag,” has been recognized as a major public health concern in Japan (1, 2). Drug lag has historically hindered timely patient access to innovative therapies. From the late 2000s to the early 2010s, discussions primarily focused on two components: “development delay,” defined as delays in regulatory submissions by sponsors, and “review delay,” defined as differences in review timelines between regulatory authorities (3). In recent years, increasing attention has been directed toward a related but distinct issue known as “drug loss” (3–5). Drug loss is defined as a situation in which a drug approved overseas is not developed for the Japanese market (6). As of March 2023, 86 products (60.1%) among drugs unapproved in Japan had already been approved in both the United States and the European Union but had not entered development in Japan. Of these, 72 products were medicines originating from emerging biopharma companies, orphan drugs, or pediatric drugs (7).

Several structural factors underlie drug lag and drug loss in Japan. These include the increasing number of products originating from emerging biopharma companies due to global shifts in the pharmaceutical industry, changes in corporate prioritization for new drug development and licensing strategies, and uncertainties in revenue projections associated with Japan's unique drug pricing system, including concerns about price reductions (1). Furthermore, Japan's share of the global pharmaceutical market has declined substantially, from approximately 25% in 1998 to 4.4% in 2023, reducing its attractiveness as a commercial market and potentially lowering the priority of development and launch strategies for Japan (8).

Separately, the global market for drug-device combination products has expanded rapidly, driven by the increasing use of biopharmaceuticals, the rise of home-based care, and growing demand for patient convenience (9). In Japan, DDDs such as PFSs, autoinjectors, multidose pen injectors, OBIs, and cartridge-based formulations have become increasingly prevalent. These include both drug-device combination products, in which the primary mode of action is pharmacological, and configurations in which the drug (e.g., a cartridge) and the medical device injection are approved and marketed separately.

DDDs that enhance usability, particularly those that facilitate self-injection, play a critical role in improving patient outcomes. They enable easier and more accurate administration, reduce dosing errors (10), and lessen the burden of clinic visits and associated costs, thereby contributing to improved quality of life and treatment adherence (11). Selecting an appropriate delivery device is therefore an important determinant of patient adherence and long-term therapeutic success (12, 30, 34).

Despite their growing importance, regulatory frameworks for combination products remain insufficiently harmonized across Japan, the United States, and the European Union (35). These differences may influence pharmaceutical companies’ development strategies and approval timelines. To our knowledge, no prior study has systematically compared market trends or approval timing for DDDs between Japan and the United States.

Japan's regulatory authorities, including PMDA, have implemented various measures to address drug lag and drug loss, such as strengthening regulatory systems, shortening review times, promoting multiregional clinical trials, and expanding fast-track pathways, including orphan drug designation and priority review (6). However, these initiatives primarily target new drug development. As the number and diversity of DDDs continue to increase, the potential for drug delivery device-related approval lag or loss remains.

In this study, we aimed to analyze approval lag and loss for DDDs, including combination products used for self-administration of biopharmaceuticals, by systematically comparing approval patterns in Japan and the United States. Through quantitative evaluation of drug delivery device lag and loss, we sought to identify regulatory challenges and provide insights to support more efficient development of DDDs.

## 2 Methods

### 2.1 Selection criteria

We examined biopharmaceutical products approved in Japan or the United States between January 2000 and December 2025, excluding small-molecule drugs, vaccines, biosimilars, and interchangeable products. Products with unclear or unverifiable approval dates in either Japan or the United States were excluded. The study population was primarily identified from biologics approved by FDA through the Biologics License Application (BLA) pathway. To capture products not represented in U.S. regulatory databases including Purple Book, biopharmaceutical products approved only in Japan by the Ministry of Health, Labour and Welfare (MHLW) were additionally included. Among these, we focused on products using DDDs, defined as PFSs, autoinjectors, multidose pen injectors, OBIs, and cartridge formulations. Cartridge products were included only when they were designed for use with a dedicated administration device sold separately. In addition, for selected brands that included these device-based presentations, vial formulations were also included for comparison.

Data were aggregated at the brand level, with multiple strengths of the same presentation counted as a single entry. For classification of small-molecule drugs, biopharmaceuticals, and vaccines, products marketed only in Japan were categorized according to Japanese regulatory definitions, whereas products marketed in both regions were classified according to U.S. regulatory categories.

### 2.2 Primary outcome measures

#### 2.2.1 Approval trends

Approval trends were assessed by evaluating five-year interval trends in the number of approvals in Japan and the United States for all DDDs and for each device category. We also assessed the proportion of each device type and differences in device configurations and components within the same brand across the two regions.

#### 2.2.2 Approval loss by drug versus device presentation

Approval loss was assessed from two perspectives. Approval loss by drug was defined as the number and proportion of products for which the active substance was approved in one country (Japan or the United States) but not in the other, regardless of device presentation. Approval loss by device presentation was defined, after excluding cases of approval loss by drug, as the number and proportion of products for which the active substance was approved in both countries but a specific device presentation was not approved in one of the regions. For vial presentations, the analysis was limited to brands that included at least one device presentation, and vials were evaluated as reference presentations. Both approval loss by drug and approval loss by device presentation were defined exclusively on the basis of regulatory approval status and did not include differences in approved dosage strengths, supply-related issues (e.g., shipment suspensions or product recalls), or commercial availability considerations (e.g., products approved but not marketed, discontinued after approval, or never launched).

#### 2.2.3 Approval lag by device presentation

Mean and median values, as well as five-year interval trends, were evaluated based on differences in approval dates. For U.S.-first products, approval lag by device presentation was calculated as the Japan approval date minus the U.S. approval date, expressed in months. For Japan-first products, approval lag by device presentation was calculated as the U.S. approval date minus the Japan approval date, expressed in months.

#### 2.2.4 Device presentation availability and timing of approval

The time from initial product approval to autoinjector approval was analyzed in each country, together with the types of device presentations introduced. In addition, the number of approved presentations per brand, including vial formulations and device presentations was compared between Japan and the United States.

### 2.3 Data sources

For Japan, the PMDA Pharmaceutical Information Search database (13), the MHLW National Health Insurance (NHI) Drug Price List (14), and the National Institute of Health Sciences (NIHS) list of approved biopharmaceuticals (15) were used. For the United States, the FDA Purple Book (16) and the FDA-Approved Drug Products database, including approval letters and labeling information (17), were referenced. To exclude vaccines not intended for self-administration, the FDA list of Vaccines Licensed for Use in the United States (18) was also consulted.

### 2.4 Statistical analysis

Descriptive statistics were used to summarize approval loss and approval lag across device presentations. Differences in the product approval between Japan and the United States were evaluated using Pearson's chi-square test. Fisher's exact test was additionally performed because of the small number of some presentation categories. Approval lag was summarized using the mean, standard deviation (SD), median, minimum, maximum, and interquartile range (IQR). Differences in approval lag among device presentations were evaluated using the Kruskal–Wallis test. All statistical tests were two-sided, and a *p*-value < 0.05 was considered statistically significant. Statistical analyses were performed using R version 4.5.3 (R Foundation for Statistical Computing, Vienna, Austria).

## 3 Results

### 3.1 Approval trends in the Japanese and U.S. markets

Between 2000 and 2025, a total of 393 products, including biopharmaceuticals and vaccines, were identified in Japan and the United States that used PFS, autoinjectors, multidose pen injectors, OBIs, cartridge formulations, or vial formulations belonging to brands that included these device-based presentations (Table 1). Across device types, PFS accounted for the largest number of approvals in both countries, followed by autoinjectors. Of these, 8 products with unclear approval dates in either country, 43 vaccines, and 81 biosimilars or interchangeable products were excluded, resulting in 261 products included in the final analysis (Figure 1).

**Table 1:** Classification and number of drug delivery device products.

Drug delivery device category	Japan	United States
Prefilled syringe (with needle or without needle)	57	77
Autoinjector^a^ [single-use, with needle, integrated syringe (excluding prefilled syringes)]	36	42
Multidose pen injector^a^ (reusable, without needle, integrated syringe)	31	35
On-body injector^a^	3	4
Cartridge (for attachment to drug delivery devices distributed separately from the drug product)	20	18
Vial presentations among products with drug delivery device presentations (dropper vial, multidose vial, single-dose vial, or vial)	40	65
Total number of products using drug delivery devices	187	241

a In the Purple Book, all such products are classified simply as “Autoinjector,” without distinguishing among autoinjectors, Multidose pen injectors, or on-body injectors.

**Figure 1:**
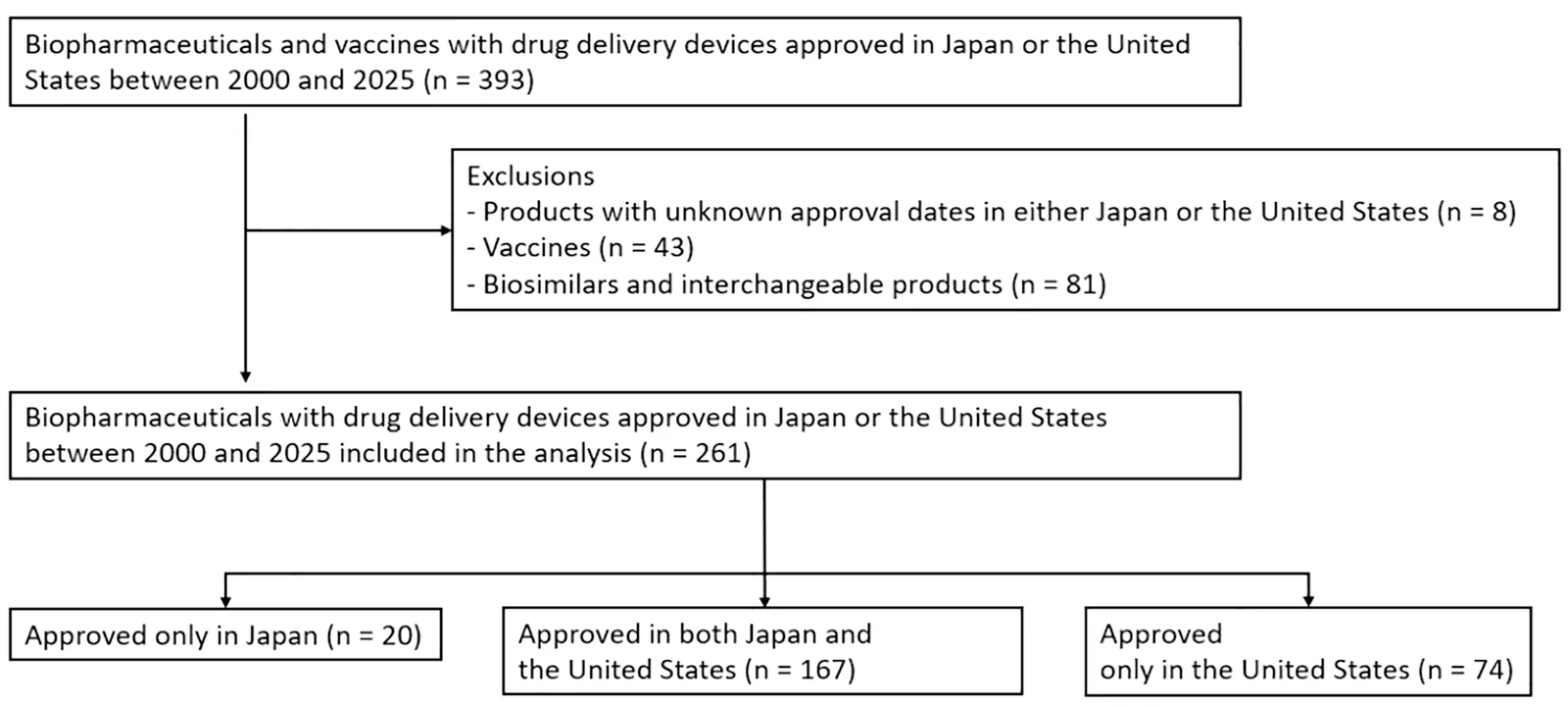
Flowchart of selection process.

Among the 261 products analyzed, 20 were approved only in Japan, 167 were approved in both Japan and the United States, and 74 were approved only in the United States (Figure 1).

Overall, the number of approved biologics with device presentations increased sharply in both Japan and the United States beginning in 2001, reflecting the rapid expansion of device-enabled biopharmaceuticals during this period. Over the study period, the number of approved products approximately doubled in the United States, whereas it increased nearly fivefold in Japan (Figure 2A).

**Figure 2:**
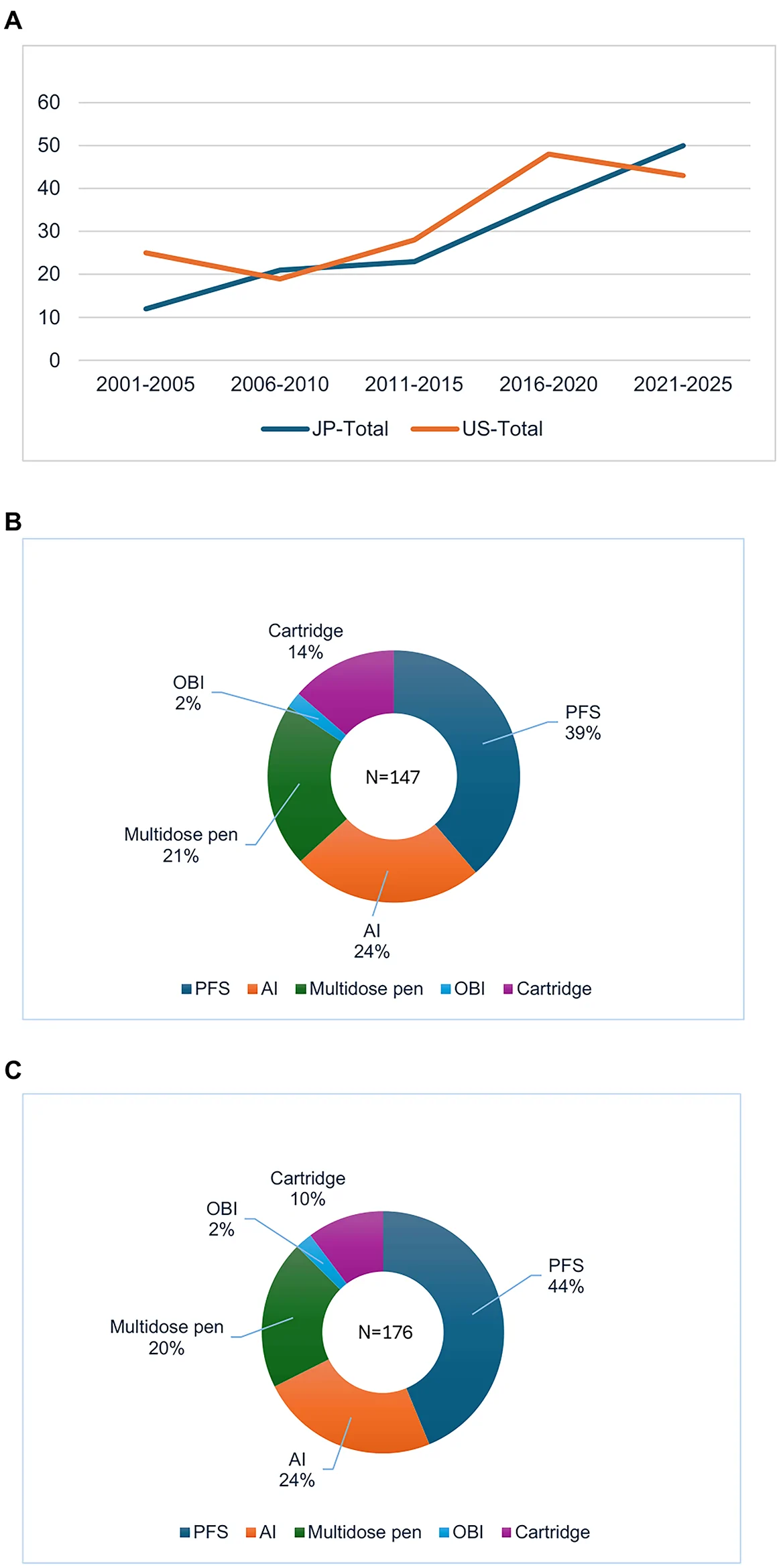
Approval trends and distribution of drug delivery device presentations in Japan and the United States. **(A)** 5-year interval approval trends by drug delivery device (Japan vs. United States). **(B)** Proportion of drug delivery device approvals (JP). **(C)** Proportion of drug delivery device approvals (US).

The analysis showed no major differences in the proportional distribution of device presentation between Japan and the United States. The United States had a slightly higher proportion of PFS, whereas the proportion of cartridge presentations was slightly lower than that in Japan. The relative proportions of autoinjectors, multidose pen injectors, and OBIs were largely comparable between the two regions (Figures 2B,C).

Among the products included in this study, 4% showed differences in drug delivery device configuration between Japan and the United States, whereas 9% showed differences in package components within the same brand (Figure S1). ration differences included the use of injection devices from different manufacturers with distinct designs and form factors, while component differences included cases in which needles were packaged with the product in the United States but not in Japan.

### 3.2 Five-year trends in approvals of drug delivery device products in Japan and the United States

Five-year interval trends in the number of approvals for DDDs between 2001 and 2025 were examined for Japan and the United States. Approvals of products using PFS and autoinjectors increased sharply over time in both countries. In contrast, approvals of multidose pen injectors, OBIs, and cartridge formulations either declined or remained relatively stable throughout the study period (Figures S2 and S3).

### 3.3 Approval loss by device presentation in the Japanese market

Among the 176 U.S. FDA-approved biologics with device presentations, excluding vial presentations, 16% were not approved in Japan (Figure 3A).

**Figure 3:**
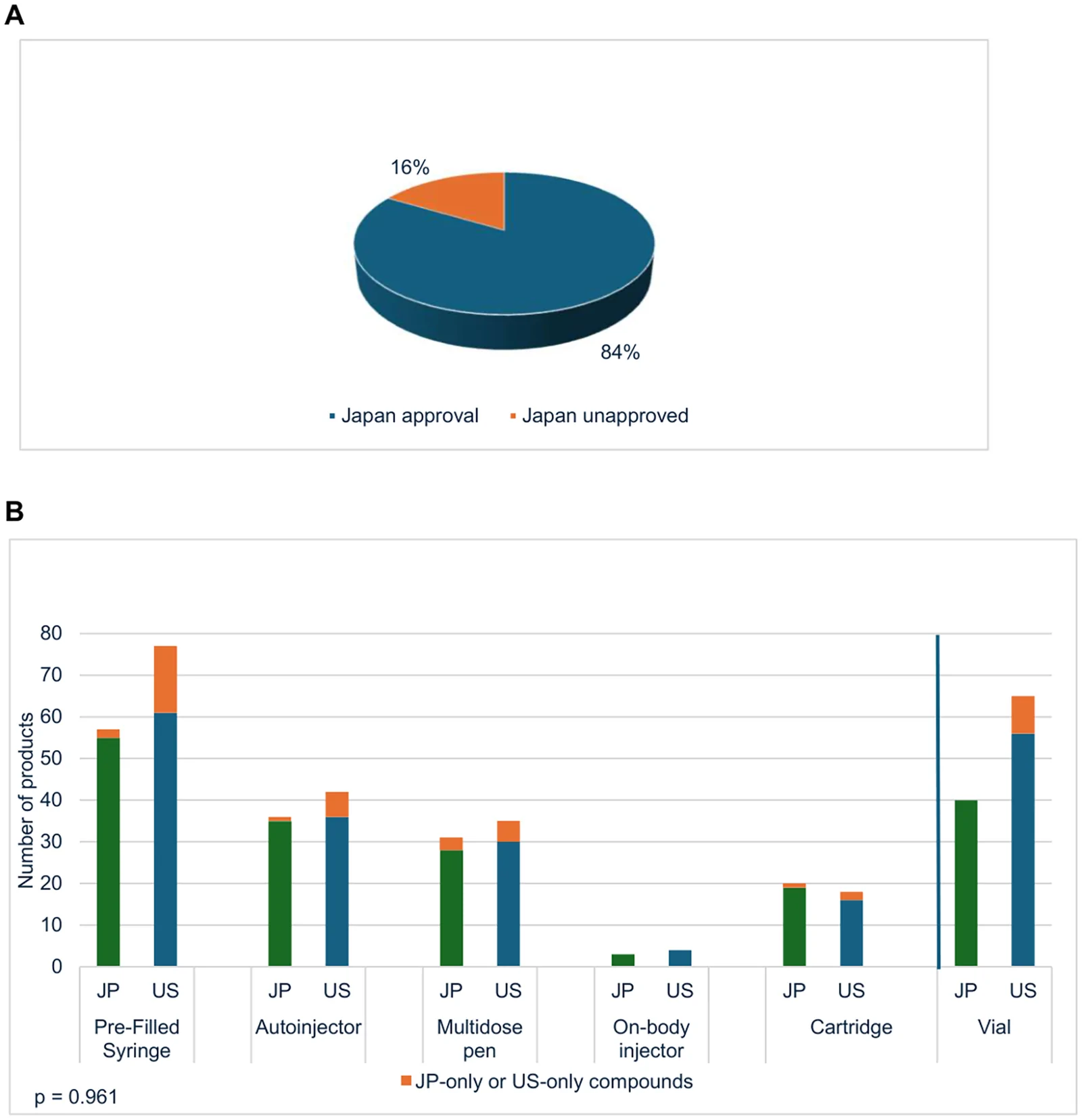
Approval loss of drug delivery device presentations in Japan among U.S.-approved biologics. **(A)** Japan approval among U.S.-approved drug delivery device (*n* = 176). **(B)** Number of products by drug delivery device in Japan and the United States.

When U.S.-approved products that remained unapproved in Japan were examined by device presentation, PFS showed the largest discrepancy in the number of approved products, followed by autoinjectors. In contrast, for cartridge formulations, the number of approved products was higher in Japan than in the United States.

However, when cases in which the active substance itself was not approved in Japan were excluded, namely products for which none of the presentations were approved regardless of device presentation, the difference in the number of drug delivery device products between the two countries was minimal. Even for PFS, which showed the largest discrepancy, only six U.S.-approved products lacked approval in Japan.

A chi-square test was conducted to evaluate differences in the approval of device presentations between Japan and the United States after excluding products for which the active substance itself was not approved in one of the countries. No statistically significant difference was observed (Pearson's *χ*^2^ = 0.62, df = 4, *p* = 0.961), indicating that the observed differences in approval numbers by device presentation were minimal and not systematic. Because of the small number of OBIs, Fisher's exact test was additionally performed and yielded a similar result (*p* = 0.963), confirming the absence of a significant association (Figure 3B).

### 3.4 Approval lag by device presentation between Japan and the United States for U.S.-first products

Differences in approval dates between Japan and the United States for U.S.-first products, stratified by device presentation, were as follows: PFS, median lag of approximately 12 months; autoinjectors, median lag of approximately 10 months, the shortest among device categories; multidose pen injectors, median lag of approximately 24 months; OBIs, median lag of approximately 14 months; and cartridge presentations, median lag of approximately 31 months, the longest among device categories.

Overall, there were 93 U.S.-first products and 34 Japan-first products, indicating that U.S.-first cases were approximately 2.7 times more common.

When examined by device presentation, multidose pen injectors and cartridge presentations showed the largest approval gaps between the two countries, with median differences exceeding approximately two years. In contrast, autoinjectors showed the smallest approval gap among the five device presentations, with a median lag of approximately 10 months for U.S.-first products (Figures 4A,B; Supplementary Table S1; Figure S4).

**Figure 4:**
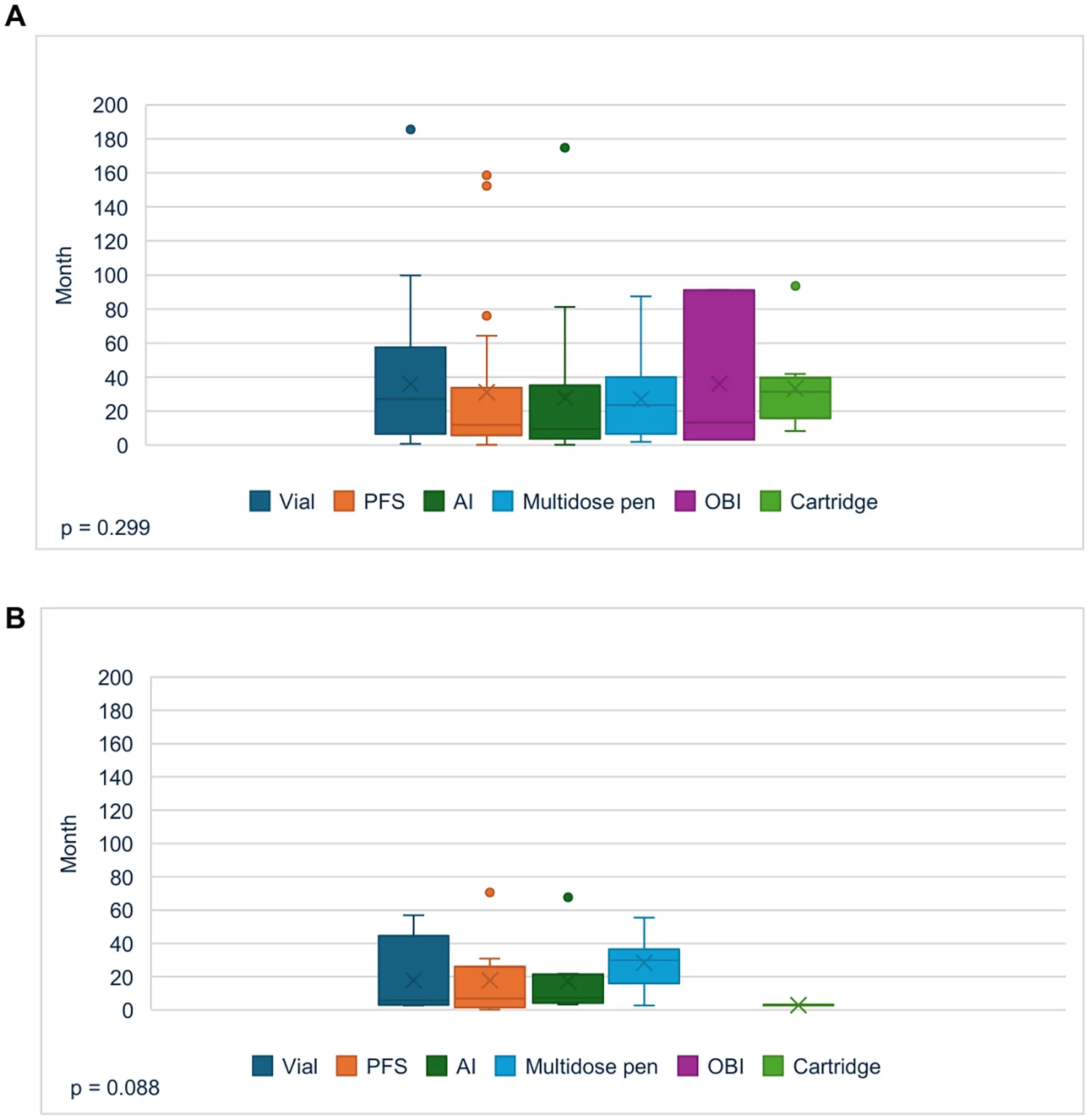
Approval lag by device presentation between Japan and the United States. **(A)** Difference in approval dates for U.S.-first products (JP − US, months). **(B)** Difference in approval dates for Japan-first products (US − JP, months).

Kruskal–Wallis tests were performed to assess differences in approval lag among device presentations after excluding vial presentations. No statistically significant differences were observed among device presentations for either Japan-first products (*χ*^2^ = 6.55, df = 3, *p* = 0.088) or U.S.-first products (*χ*^2^ = 4.89, df = 4, *p* = 0.299). These findings indicate that, although median approval lag differed numerically across device presentations, the observed differences were not statistically significant. OBI was excluded from the Japan-first analysis because no observations were available.

### 3.5 Time from initial product approval to approval of autoinjector presentations

Given that autoinjectors showed the smallest approval lag between Japan and the United States in our analysis, we further examined the time from first approval of a brand to subsequent approval of its autoinjector presentation in both countries. When brands for which the autoinjector was the only approved presentation were included, approximately half of all products in both Japan and the United States obtained autoinjector approval within one year of initial product approval.

However, the composition of these “within one year” cases differed markedly between the two countries. In the United States, autoinjector presentations were frequently approved simultaneously with other presentations at the time of initial approval, reflecting a strategy of securing multiple device presentations upfront. In contrast, Japan showed a substantially higher proportion, approximately twice that of the United States, of products for which the autoinjector presentation was approved as a standalone presentation.

Additionally, the proportion of products requiring more than eight years from initial approval to autoinjector approval was higher in the United States than in Japan (Figures 5A,B).

**Figure 5:**
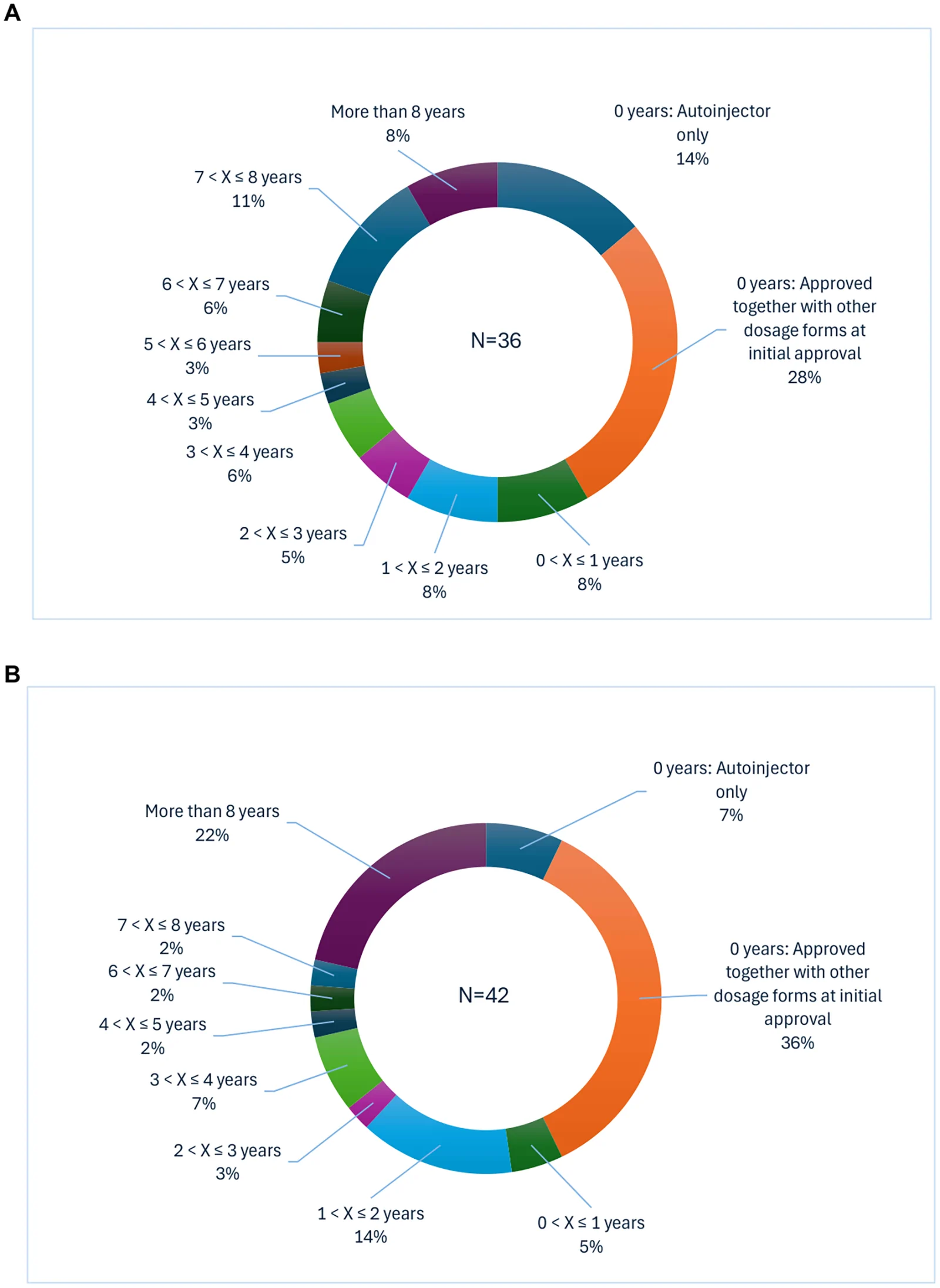
Time from initial product approval to approval of autoinjector presentations. **(A)** Years from initial approval to autoinjector approval in Japan. **(B)** Years from initial approval to autoinjector approval in United States.

### 3.6 Approval status of device presentations

#### 3.6.1 Approval patterns of brands containing autoinjector presentations

The approval status of other presentation types in Japan and the United States was examined for brands that included an autoinjector presentation. In both countries, the most common combination was autoinjector plus PFS, followed by autoinjector plus PFS plus vial. The proportion of brands with the autoinjector plus PFS combination was nearly identical between Japan and the United States, accounting for approximately half of all such brands in each market.

In contrast, Japan had a substantially higher proportion, approximately twice that of the United States, of brands for which the autoinjector was the only approved presentation. Conversely, the United States had a higher proportion of brands approved with the autoinjector plus PFS plus vial combination, which was approximately 1.5 times more frequent than in Japan (Figures 6A,B).

**Figure 6:**
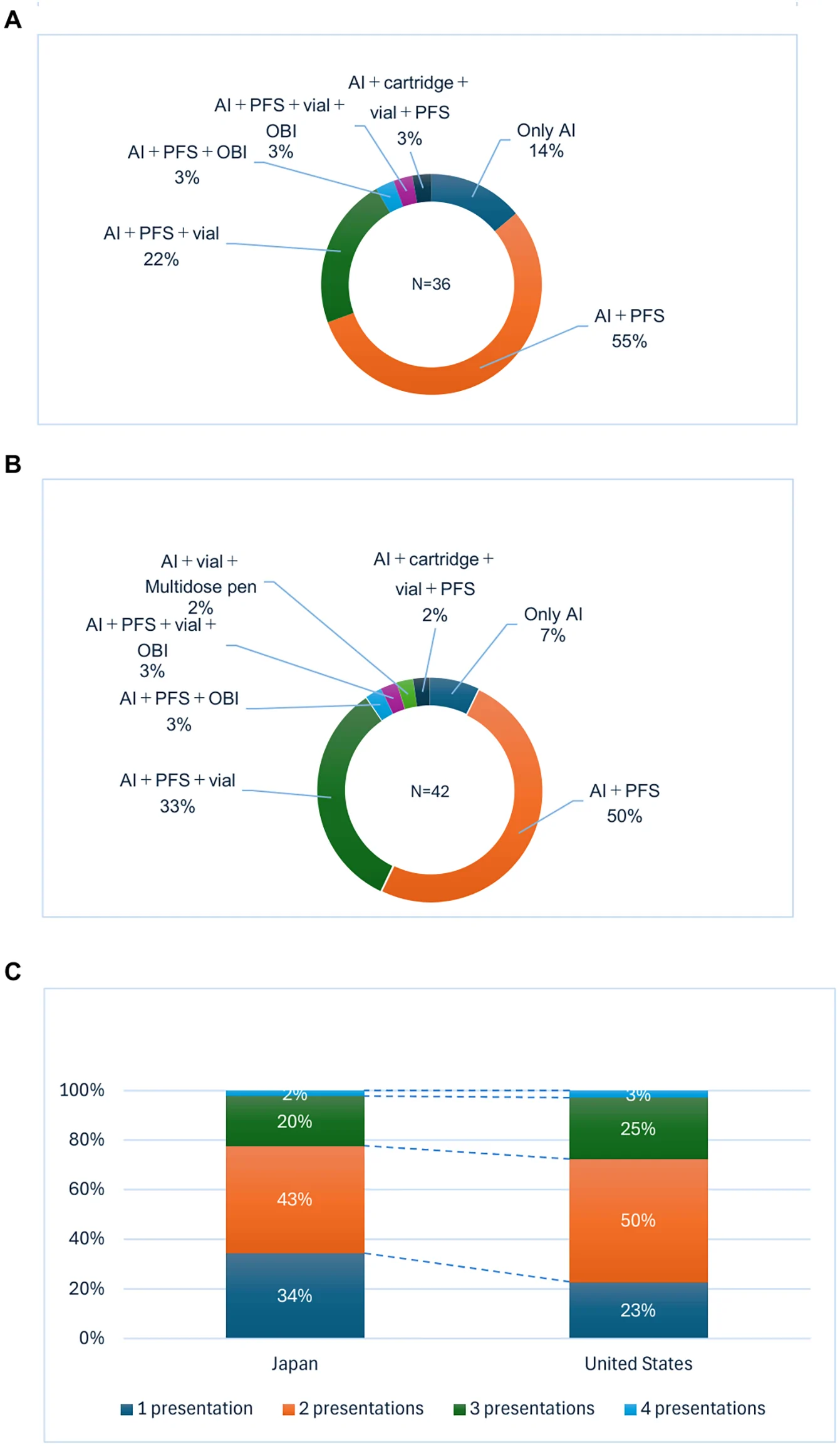
Availability and approval patterns of autoinjector presentations. **(A)** JP: Types of presentations among brands with autoinjector formulations. **(B)** US: Types of presentations among brands with autoinjector formulations. **(C)** Number and proportion of presentations in Japan and the United States.

#### 3.6.2 Comparison of multiple-presentation approvals in Japan and the United States

We compared the number of approved formulations, including vial presentation and device presentations, per brand in Japan and the United States. In both countries, two presentations represented the most common approval pattern. However, 77% of brands in the United States had obtained approval for multiple presentations, whereas only 66% of brands in Japan had approvals for more than one presentation. The remaining 34% of brands in Japan were approved with only a single presentation (Figure 6C).

## 4 Discussion

### 4.1 Limited approval loss by device between Japan and the United States

Our analysis of market and approval trends for biologics with Device presentations in Japan and the United States revealed a consistent increase in the number of approved products in both regions.

Over the past decade, approvals of OBIs and autoinjectors have gradually increased, reflecting the diversification of drug delivery device technologies. This expansion is consistent with broader trends in biopharmaceutical development, including the shift toward self-administration, patient-centric design, and growing demand for devices that enhance usability, safety, and adherence. However, despite the continuous introduction of diverse novel DDDs, the difference in the number of approved device presentations between Japan and the United States was markedly reduced, with little variation observed among device presentation types. This indicates that although approval loss attributable to the drug remains a significant issue in Japan, the impact of approval loss by device is relatively limited.

### 4.2 Device-specific characteristics and underlying factors contributing to approval lag by device

Our analysis of approval date differences between Japan and the United States revealed substantial variation in approval lag across device presentation types. Although the differences were not statistically significant, multidose pen injectors and cartridge presentations showed the largest discrepancies, with median approval gaps exceeding approximately two years for U.S.-first products.

In addition to drug lag, several factors related to both development delays and regulatory review delays have been identified as contributors to approval lag by device. Factors associated with development delays include requirements for Japan-specific nonclinical data, the administrative burden associated with the translation and preparation of regulatory documentation, delays in obtaining necessary data from overseas headquarters, and internal decision-making regarding whether and when to pursue a regulatory submission in Japan (19–21).

With respect to regulatory review delays, part of the extended lag observed for multidose pen injectors and cartridge presentations may be attributable to the fact that, in addition to pharmaceutical approval, some of these products had historically required approval or certification as medical devices in Japan. For such products, medical device approval or certification typically preceded pharmaceutical marketing authorization, potentially prolonging the overall regulatory timeline. However, following the issuance of the 2014 regulatory notification on combination products in Japan (22), when the primary mode of action is pharmaceutical and the product falls within the scope of the combination product guidance, dual submission as both a pharmaceutical and a medical device is no longer required. As a result, particularly for integrated pen injectors, many recent submissions have proceeded solely under the pharmaceutical regulatory pathway, likely contributing to shorter overall review times.

Furthermore, many of the multidose pen injector and cartridge presentation approvals included in our dataset occurred between 2006 and 2010, a period corresponding to the early phase of national discussions on drug lag in Japan (23, 24), before comprehensive countermeasures had been implemented. During this period, review timelines were generally longer than they are today. In recent years, regulatory reforms have accelerated review processes, and approval timelines for both pharmaceuticals and medical devices in Japan have approached those of the FDA and EMA (25–27).

In contrast, autoinjectors demonstrated the smallest approval lag between Japan and the United States. This device type has shown a rapid increase in approvals since 2015 (28), coinciding with the implementation of combination product guidance and broader drug lag mitigation efforts. In typical development strategies, device development proceeds in parallel with clinical trials, with initial launches often relying on basic vial or syringe presentations, followed by sequential introduction of PFS or autoinjector presentations. This staged approach has historically been used to manage technical challenges and reduce development risk. However, as DDDs have become more common and regulatory pathways more established, biopharmaceutical companies have increasingly invested in device development from the early stages, contributing to shorter delays in bringing advanced devices to market (29). Furthermore, our findings showed that Japan had a higher proportion of products approved with autoinjector-only presentations than the United States. This suggests that some sponsors may be prioritizing direct development of autoinjector presentations rather than adopting a stepwise approach from vial to PFS and subsequently to autoinjectors. Such a strategy may facilitate faster market introduction and could have contributed to the relatively small approval lag observed for autoinjector presentations between the two countries.

### 4.3 Differences in approval strategies between Japan and the United States: multi-presentation deployment at initial approval

When multi-presentation approval patterns for biopharmaceuticals were examined, the proportion of single-presentation approvals in Japan was approximately 1.5 times higher than that in the United States. This indicates that although both countries strategically deploy presentations intended for clinical settings, such as PFS and vial presentations, and those intended for self-administration, such as autoinjectors, their approaches at the time of initial approval differ substantially.

In the United States, multiple presentations, including vial presentations, were commonly approved simultaneously at initial approval. This suggests that pharmaceutical companies more frequently submit applications containing a full suite of presentations from the outset. Such an approach enables healthcare providers to tailor treatment to patient-specific needs, preferences, and financial considerations, potentially improving long-term adherence (30, 31).

In contrast, Japan more commonly adopts a stepwise strategy, in which basic presentations, such as PFS or vials, are approved first, followed by the introduction of additional device presentations at later stages. Several factors may contribute to this pattern, including cautious assessment of return on investment in the Japanese market, established clinical practice patterns, challenges associated with adopting multiple presentations within healthcare institutions (32), and administrative burdens related to maintenance and withdrawal of marketing approvals.

### 4.4 Factors underlying Japan-specific combination product packaging and policy implications

In this study, 9% of products showed differences in the components included in the product package between Japan and the United States. Although some of these differences may reflect company-specific strategies to tailor device components to individual markets, a more fundamental driver is the divergence in regulatory frameworks across countries. These regulatory differences can necessitate variations in package contents, such as whether certain device components, including needles or administration syringes, can be included within a single product package, even for products that are otherwise identical.

In particular, Japan and the United States differ in their definitions and regulatory scope of combination products, as well as in the allowable range of components that may be included within a single product submission. As a result, supplying an identical package to both markets is often not feasible, and companies may be required to prepare Japan-specific packages with unique component configurations. Such additional packaging requirements can prolong pre-submission preparation and may contribute to approval lag between the two countries. Moreover, after approval, country-specific packaging increases supply chain complexity (33), posing risks not only to operational efficiency but also to stable product supply.

As the device components of combination products continue to advance and diversify rapidly, these challenges are likely to intensify. In this context, international harmonization of regulatory concepts and scope for combination products, including alignment on component requirements and packaging standards, will be essential. Such harmonization would support more efficient product introduction, streamline regulatory review, and enhance supply chain robustness, ultimately contributing to improved patient access and public health outcomes.

## 5 Conclusion

This study conducted the first systematic and quantitative comparison of approval patterns for biopharmaceuticals using DDDs in Japan and the United States and clarified the extent of approval lag and loss by device between the two countries. Three major findings were identified.

Approximately 16% of device presentation approved in the United States were not approved in Japan. However, most of these cases were attributable not to differences in device availability but to “approval loss by drug,” defined as cases in which the active substance itself had not been approved in Japan, regardless of device presentation. This indicates that approval loss by device should be understood primarily as a subset of approval loss by drug, with active substance approval status being the dominant determinant.

Second, comparison of approval dates revealed substantial variation in approval lag across device presentation types. Multidose pen injectors and cartridge presentations exhibited numerically longer median approval lags, each exceeding approximately two years, whereas autoinjectors demonstrated the shortest median lag. However, these differences among device presentations were not statistically significant. The observed numerical differences may reflect multiple factors, including pre-submission burdens, historical requirements for medical device approval or certification, the regulatory environment at the time of submission, and evolving corporate submission strategies.

Third, clear differences were observed in approval strategies between the two countries. In the United States, multiple presentations were frequently approved simultaneously at initial approval, supported by a regulatory and market environment that facilitates self-administration and maximizes user choice. In Japan, however, a stepwise approach was more common, with basic presentations approved first and additional device presentations introduced later. This pattern may be influenced by market size, return-on-investment considerations, and clinical practice norms. Additionally, some products showed differences in components between Japan and the United States, reflecting regulatory discrepancies that necessitate Japan-specific packaging. Such requirements may prolong preparation for regulatory submission and introduce risks to supply chain stability.

Taken together, these findings demonstrate that differences in approval outcomes for DDDs between Japan and the United States arise not only from review timelines but also from a complex interplay of regulatory frameworks, approval strategies, market structures, and institutional environments. Moving forward, accelerating compound approval and promoting multi-presentation strategies from the initial submission stage will be important for reducing device lag. Moreover, as DDDs continue to advance and diversify, international harmonization of regulatory concepts and scope for combination products will be essential for minimizing approval delays, expanding user choice, and ensuring stable product supply.

This study provides the first systematic quantitative assessment of Japan-U.S. differences in DDDs approvals including biologics-device products. Future research incorporating detailed qualitative analyses, such as review documents, company submission strategies, and records of pre-submission consultations with regulatory authorities, will be valuable for further elucidating the underlying drivers of approval lag by device.

## 6 Limitations

This study has several limitations. First, the analysis focused primarily on combination products and biopharmaceuticals listed in the Purple Book. As a result, DDDs used for small-molecule drugs and vaccines were not included, and the findings do not fully capture trends across all drug delivery device categories. Second, the study was based on products that were approved at the time of data collection; therefore, products that had already been withdrawn or had specific device presentations delisted may not have been fully identified. Third, the definition of approval loss by device was based on products that were unapproved at the time of the survey. Consequently, some products categorized as “unapproved” may in fact represent future device lag, as they could be under development or pending regulatory submission.

## Data Availability

The original contributions presented in the study are included in the article/Supplementary Material, further inquiries can be directed to the corresponding author.

## Supplementary material

SUPPLEMENTARY FIGURE S1 Differences in device configurations and package components.

SUPPLEMENTARY FIGURE S2 Japan: 5-year interval approval trends by drug delivery device.

SUPPLEMENTARY FIGURE S3 United States: 5-year interval approval trends by drug delivery device.

SUPPLEMENTARY FIGURE S4 Drug delivery device lag among U.S.-first products over time.

SUPPLEMENTARY TABLE S1 Detailed descriptive statistics for approval lag.
