# Supplementary material for: Approval lag and loss for biologic-device combination products in Japan and the United States
Source: Front Med Technol. 2026 Sep 18;8:1924169. doi: 10.3389/fmedt.2026.1924169 (PMC13630725; doi:10.3389/fmedt.2026.1924169)
Supplement: Supplementary file 1 [file Table1.pdf]

**A)** Approval date difference for U.S.-first products (Japan − United States, months)						
Statistic	Vial	PFS	AI	Multidose pen	OBI	Cartridge
N	32	35	24	19	3	12
Mean	36.1	31.1	27.9	27.0	36.0	33.3
SD	38.3	43.4	40.1	23.3	48.0	22.3
Median	27.2	11.9	9.5	23.7	13.5	31.3
Min	0.8	0.2	0.2	1.9	3.4	8.4
Max	185.6	161.5	174.9	87.6	91.1	93.6
IQR	45.3	26.5	26.2	32.7	43.9	16.8
**B)** Approval date difference for Japan-first products (United States − Japan, months)						
Statistic	Vial	PFS	AI	Multidose pen	OBI	Cartridge
N	4	15	8	9	0	2
Mean	17.8	17.7	17.2	28.4	N/A	2.9
SD	26.2	24.3	21.7	15.3	N/A	0.3
Median	5.7	7.0	7.2	29.9	N/A	2.9
Min	2.7	0.3	3.4	2.7	N/A	2.7
Max	56.9	75.0	67.8	55.6	N/A	3.2
IQR	15.4	21.2	15.6	18.6	N/A	0.2
